# Inhibiting nighttime melatonin and boosting cortisol increase patrolling monocytes, phagocytosis, and myelination in a murine model of multiple sclerosis

**DOI:** 10.1038/s12276-023-00925-1

**Published:** 2023-01-13

**Authors:** Majid Ghareghani, Vincent Pons, Nataly Laflamme, Kazem Zibara, Serge Rivest

**Affiliations:** 1 Neuroscience Laboratory, CHU de Québec Research Center, Department of Molecular Medicine, Faculty of Medicine, Laval University, 2705 Laurier Boul., Québec City, QC G1V 4G2 Canada; 2 PRASE and Biology Department, Faculty of Sciences-I, Lebanese University, Beirut, Lebanon

**Keywords:** Neuroimmunology, Multiple sclerosis

## Abstract

Conflicting results on melatonin synthesis in multiple sclerosis (MS) have been reported due to variabilities in patient lifestyles, which are not considered when supplementing melatonin. Since melatonin acts through its receptors, we identified melatonin receptors in oligodendrocytes (OLs) in the corpus callosum, where demyelination occurs; the subventricular zone, where neural stem/progenitor cells (NSPCs) are located; and the choroid plexus, which functions as a blood-cerebrospinal fluid barrier. Moreover, using chimeric mice, resident macrophages were found to express melatonin receptors, whereas bone marrow-derived macrophages lost this expression in the demyelinated brain. Next, we showed that cuprizone-fed mice, which is an MS model, tended to have increased melatonin levels. While we used different approaches to alter the circadian rhythm of melatonin and cortisol, only the constant light approach increased NSPC proliferation and differentiation to oligodendrocyte precursor cells (OPCs), OPCs maturation to OLs and recruitment to the site of demyelination, the number of patrolling monocytes, and phagocytosis. In contrast, constant darkness and exogenous melatonin exacerbated these events and amplified monocyte infiltration. Therefore, melatonin should not be considered a universal remedy, as is currently claimed. Our data emphasize the importance of monitoring melatonin/cortisol oscillations in each MS patient by considering diet and lifestyle to avoid melatonin overdose.

## Multiple sclerosis: Understanding the role of melatonin

Regulating melatonin levels holds promise as a therapy for multiple sclerosis (MS), but treatment must be tailored to each individual. In MS, the immune system destroys the myelin sheath surrounding nerves, disturbing brain–body communication. Melatonin, a sleep-inducing hormone, is known to play a role in MS, but results so far are inconsistent. Serge Rivest at Laval University in Québec City, Canada, and coworkers investigated how melatonin affects particular brain cell types in a mouse model. They found that maintaining melatonin levels at an intermediate level, where they regulate rather than enhance immune activity, could minimize myelin destruction in MS. They conclude that melatonin should not be considered a universal remedy for MS, but that melatonin levels should be monitored for each individual patient to account for lifestyle differences.

## Introduction

Since the discovery of melatonin (N-acetyl-5-methoxytryptamine) in 1958 by Aaron B. Lerner and colleagues, a substantial number of investigations have been carried out to elucidate its source, pattern of synthesis, and physiological and pathophysiological functions. Melatonin is mainly synthesized by the pineal gland in a circadian-dependent manner. Its secretion starts gradually at onset of the dark phase at night, peaks at midnight and declines by sunrise. However, any dim to bright light at night significantly affects the secretion pattern of melatonin by delaying peak secretion or shortening the secretion period^1^. Moreover, melatonin is released by other sources in a circadian-independent manner, such as the gastrointestinal tract. Several studies have investigated the role of melatonin in the pathophysiology of multiple sclerosis (MS), a chronic disease of the central nervous system (CNS). In fact, MS is suspected to result from an autoimmune attack leading to the demyelination of nerve fibers and progressive neurodegeneration. One of the pioneering studies on the role of melatonin in MS was carried out by Constantinescu et al.^2^ in 1997, whose findings implicated the deleterious role of melatonin in experimental autoimmune encephalomyelitis (EAE). EAE mice treated with luzindole, an antagonist of melatonin receptors, did not develop EAE; thus, the authors concluded that inhibiting melatonin could be used as a therapeutic strategy. However, later studies reported conflicting results. For instance, Kang et al. in 2001, showed that the inhibitory effect of melatonin on EAE mice was mediated by the suppression of intercellular adhesion molecule-1 (ICAM-1)^3^. On the other hand, Álvarez-Sánchez et al.^4^ and Chen et al.^5^ reported the beneficial role of melatonin by altering the T effector/regulatory balance, enhancing interleukin-10 (IL10) expression and suppressing chemotaxis. These neuroprotective effects of melatonin were further demonstrated by Wen et al.^6^ in 2016 and Long et al.^7^ in 2018.

Our previous results showed that melatonin was reduced in EAE mice, and that melatonin therapy improved the severity of the disease but not in younger mice^8–10^. In addition, we showed that melatonin therapy reduced the risk of osteoporosis and normalized bone formation in EAE mice^9^. Moreover, the administration of a muscle relaxant before melatonin therapy improved the efficacy of melatonin in the EAE model since the peals in melatonin were concomitant with the time at which the muscles were resting and the body was exerting its antioxidant activity^10^. Furthermore, the amelioration of EAE by corticosteroid therapy was shown to be associated with a reduction in endogenous melatonin levels^11^. At the metabolic level, melatonin treatment of EAE was linked to the inhibition of pyruvate dehydrogenase complex (PDC) activity by pyruvate dehydrogenase kinase 4 (PDK4), a key enzyme in fatty acid synthesis during the remyelination process^12^. In addition, combination treatment with melatonin and the PDK4 inhibitor diisopropylamine dichloroacetate (DADA) eliminated this side effect^13^. Overall, these studies highlighted that the previous conflicting results were in part due to differences in age, sex, dose and timing of melatonin administration in the various MS models. In addition to EAE, the cuprizone (CPZ) demyelination model showed contradictory results. In fact, Labunets and Rodnichenko revealed that melatonin therapy improved neurogenesis in CPZ mice^14^. In contrast, in 2015, Vakilzadeh et al. reported a significant increase in nuclear factor kappa B (NF-κB) in CPZ-fed mice, which directly correlated with oligodendrocyte (OL) death and was further amplified by melatonin therapy without any remyelination improvement^15^.

On the other hand, melatonin is released into the third ventricle from the pineal gland and interacts with ependymal cilia of the subventricular zone (SVZ), where neural stem/progenitor cells (NSPCs) are located^16^. Although NSPC differentiation and maturation lead to OL formation, their function in adults is poorly understood. In mice, NSPCs were shown to migrate to the olfactory bulb to produce new neurons and to the site of injury to produce new glial cells, including astrocytes and OLs^17^.

Overall, due to the conflicting results and lack of a mechanistic pathway by which melatonin affects MS, we aimed to investigate the effect of manipulating endogenous and exogenous melatonin levels on the activation or inhibition of melatonin receptors and on NSPC activation and differentiation to OLs. We also aimed to study the expression of melatonin receptors in different regions of the brain and investigate their role in monocyte phenotypes, infiltration, and phagocytosis.

## Material and Methods

### Antibodies and products

Specifications of the antibodies used in this study for tissue immunofluorescent staining and for fluorescence‐activated cell sorting (FACS) analysis are listed in Supplementary Table 1. All other reagents are listed in Supplementary Table 2.

### Mice

Adult 12-week-old male C57BL/6 J wild-type mice were used for the 3-week treatment protocol. We also used adult male C57BL/6 J wild-type and sex-matched CX3CR1-GFP mice to generate chimeric mice for the 1-week treatment protocol. Experimental animal protocols complied with all relevant ethical regulations, were performed in accordance with the Canadian Council on Animal Care guidelines and were administered by the Laval University Animal Welfare Committee. All animals were acclimatized to standard laboratory conditions (12 h light, 10 h dark cycle; lights ON at 07:00 and OFF at 19:00 h) with free access to rodent chow and water.

### Demyelination model

To induce demyelination, cuprizone (0.2% wt/wt) was incorporated into standard irradiated ground rodent chow. Wild-type or chimeric mice were fed normal chow as controls or cuprizone-supplemented ground chow over the course of 5 weeks. The chow container was checked every 2 days, and uneaten chow was removed and replenished with fresh chow. Body weight was monitored once per week, and the loss of more than 15% body weight was considered exclusion criterion in the experiment.

### Experimental groups

The mice were randomly allocated to 6 groups as follows: (A) control mice fed with normal chow (Ctrl); (B) cuprizone mice treated with vehicle DMSO/Oil (Vhl); (C) cuprizone mice maintained in constant light (CL); (D) cuprizone mice maintained in constant darkness (CD); (E) cuprizone mice treated with 30 mg/kg Luzindole (LUZ); and (F) cuprizone mice treated with 80 mg/kg melatonin (MLT). All treatments (LUZ and MLT) and light manipulations were initiated at the beginning of the 3rd or 5th week and were performed on wild-type mice for 3 weeks or on chimeric mice for 1 week, respectively.

**Fig. 1:**
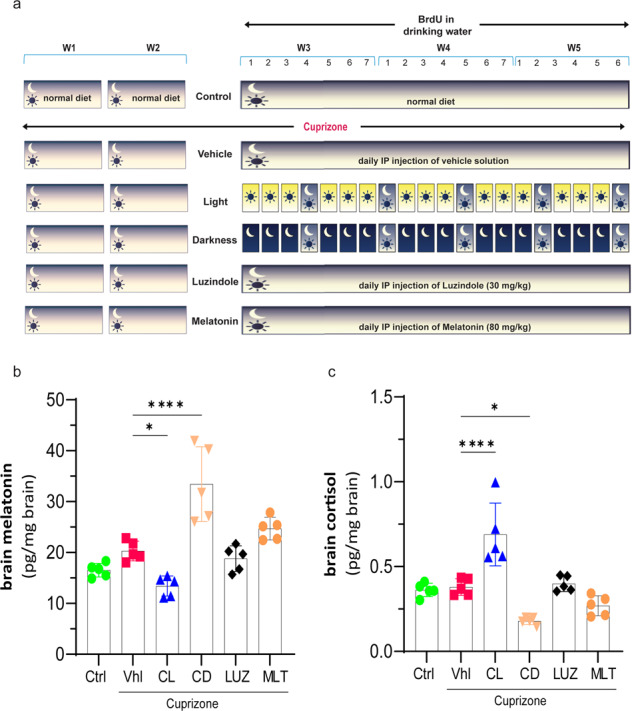
Schematic representation of the treatment protocols. **a** Briefly, the mice were fed CPZ for 2 weeks under a normal light/night cycle. Thereafter, treatments were initiated at the beginning of Week 3 in the presence of CPZ. Both the Ctrl group and the vehicle (untreated CPZ) were kept on a normal light/night cycle. The melatonin (80 mg/kg) and Luzindole (30 mg/kg) groups received these drugs by IP daily at 9 AM and 6 PM, respectively, and were maintained on a normal light/night cycle. Light was set ON or OFF for 3 days in the constant light or constant darkness groups, respectively, followed by one day of normal light/night. This cycle was repeated 5 times. BrdU in drinking water was started at the beginning of the manipulations in Week 3. **b** Brain levels of melatonin and (**c**) cortisol were analyzed using ELISA at the end of the study (*n* = 5 mice/group). Significance is indicated by ^∗^*p* < 0.05, ^∗∗^*p* < 0.01, and ^∗∗∗∗^*p* < 0.0001.

The dose of luzindole (30 mg/kg) was selected based on its effect on suppressing the severity of EAE in mice, as previously described^2^. Luzindole was administered intraperitoneally at 6 PM to inhibit melatonin receptors at night. The dose of melatonin (80 mg/kg) was selected based on its efficacy in ameliorating the severity of EAE without any toxicity^4^. Melatonin was administered intraperitoneally at 9–10 AM, when melatonin is at its lowest level, since melatonin is normally high at night^18,19^. In fact, this protocol is designed to result in high melatonin levels during the day. Luzindole and melatonin were freshly dissolved in 5% dimethyl sulfoxide (DMSO) and diluted in corn oil before each injection. The vehicle (untreated CPZ mice) and experimental groups received the same dose of 5% DMSO and corn oil. On the other hand, to manipulate the light and dark periods, mice were transferred into a “Red LEDDY Cage”, which has a fully programmable IVC lighting control system for circadian rhythm studies (Tecniplast, Italy). The mice were exposed to constant light (24 h/day bright light) at an intensity of 80 lux or constant darkness (24 h/day darkness) for 3 days, followed by a rest day of normal 12/12 h light/dark. Briefly, the mice were kept for 3 days under constant light or constant darkness to suppress or increase pineal-dependent melatonin synthesis, respectively, and 3 days was the optimal time to reach this aim. This was followed by one day of recovery to allow the mice to re-establish a normal day of 12 h light and 12 h dark. to minimize the pathological situation that may arise by keeping mice in a continuous light or dark environment. This cycle was then repeated 5 times. The experimental procedures and the various groups are summarized in a schematic representation in Fig. 1a.

### Chimeric mice

Bone marrow (BM) was extracted from sex-matched CX3CR1-GFP donors and transplanted into 8-week-old recipient male C57BL/6 J wild-type mice, which had been ablated earlier. Briefly, antibiotic therapy (Septra, 100 mg/ml drinking water) was administered to recipient mice for 7 days, followed by busulfan chemotherapy (10 mg/kg; two IP injections at 12 h intervals) for 4 consecutive days and then IP injections of cyclophosphamide (100 mg/kg; once per day) for two days. At 48 h after the last cyclophosphamide injection, the BM cells of donor mice were extracted, and approximately 8 × 10^6^ cells were intravenously transferred to each recipient mouse. To validate chimerism, peripheral blood samples were assessed by FACS to quantify the number of donor cells in recipient mice at 8 weeks post transplantation. Mice with more than 94% circulating donor cells were considered chimeric. The protocol is summarized in Fig. 7a.

### BrdU incorporation and detection

To detect the regeneration of oligodendrocyte precursor cells (OPCs) and mature OLs, the thymidine analog 5-bromo-2’-deoxyuridine (BrdU) was dissolved in a sterile 0.9% NaCl solution and added to drinking water at a concentration of 1 mg/ml. BrdU was administered at the beginning of all treatments (LUZ and MLT) and light manipulations (CL and CD) in Week 3 of the cuprizone diet and was replaced every two days with a freshly prepared solution. The volume of BrdU-supplemented water consumed was checked every two days to ensure that all mice received the same amount of BrdU.

### Sacrifice

All mice were deeply anesthetized with ketamine/xylazine (75 mg/kg versus 8 mg/kg, ip) prior to transcardial perfusion with 0.9% saline and 4% paraformaldehyde (PFA; pH 7.4). Brain tissues were collected and postfixed by immersion in the same fixative at 4 °C overnight and then stored in 20% sucrose supplemented with PFA (pH 7.4) for 12 h. The brains were then cut into 25 μm-thick sections with a freezing microtome (Leica Microsystems), serially collected in antifreeze solution and stored at −20 °C until further processing. For ELISA, brains from another set of experiments were collected quickly after transcardial perfusion with 0.9% saline and stored at −80 °C until further processing.

### Detection of brain cortisol and melatonin

Although the synthesis of cortisol in mice is controversial and it was thought that mice do not produce appreciable cortisol, Gong et al. used high-performance liquid chromatography (HPLC), electrospray ionization mass spectrometry (ESIMS), and ELISA and showed that the dynamics of cortisol and corticosterone secretion in mice closely correlated with pathological conditions, including stress^20^. This finding was further confirmed by subsequent studies^21–23^. Furthermore, Reiter et al. studied the cortisol level in C57BL/6 J mice in collaboration with Charles River Research Animal Diagnostic Services^24^. We used a mouse ELISA kit that has high sensitivity and excellent specificity for mouse cortisol, and no cross-reactivity or interference between mouse cortisol and analogs has been observed.

Briefly, the mice were anesthetized, and the brains were quickly extracted without perfusion or fixation (within 1 h) and stored at −80 °C for further studies. Thereafter, brain samples were halogenated, and the protein concentration in each sample was calculated by BCA (bicinchoninic acid) colorimetric protein measurement. Cortisol and melatonin concentrations were measured according to the kit protocol, and the values are reported in mg of brain.

### Fluorescence‐activated cell sorting (FACS) analysis

Blood was obtained from the submandibular vein before the mice were perfused, and samples were collected in heparin-coated tubes. Since the interval between sample preparation and analysis is a critical parameter for confounding factors in functional immunological assays, we carried out FACS analysis within 1 h. Briefly, erythrocytes were lysed using ACK lysis solution, and then leukocytes were washed twice by centrifugation at 500 × g. The cells were then incubated for 15 min on ice with purified rat anti–mouse CD16/CD32 antibodies and washed by centrifugation at 500 × g. Next, the cells were incubated with a cocktail of fluorescently conjugated CD45, CD11b, Ly6C, and Ly6G antibodies and Live Dead Blue fluorescent dye for 30 min. Then, the cells were washed and resuspended in PBS containing 123 counts of eBeads, and FACS analysis was performed. The results were analyzed by FlowJo software (v10.0.7).

### Immunostaining

Brain sections were washed in KPBS and then pretreated with blocking solution containing 1% (v/v) bovine serum albumin (BSA), 4% appropriate serum, and 0.4% (v/v) Triton X-100 in KPBS for 2 h. Thereafter, primary antibodies (Olig2, 1/1000; APC, 1/1000; CD68, 1/1000; Caspase-3, 1:500; SOX2, 1/1000; Ki67, 1/1000; BrdU, 1/1000; MT1A, 1/200; MT1B, 1/200) were added to the sections in 1:2 diluted blocking buffer with KPBS and incubated overnight at 4 °C, except for MT1A and MT1B, which were incubated for 48 h. Next, the sections were washed and incubated with corresponding secondary antibodies for 2 hours at room temperature. The sections were counterstained with DAPI (Sigma‒Aldrich), mounted onto MicroSlides Superforst® and cover slipped with Fluoromount-G. HCl treatment (2 N HCl, 30 min at 37 °C) and subsequent naturalization with butyric acid for 10 min were performed before blocking for BrdU staining. Twenty minutes of antigen retrieval with 95-degree Celsius Sodium Citrate Buffer (10 mM sodium citrate, 0.05% Tween 20, pH 6.0) was performed for SOX2 and Caspase-3 antibodies before blocking.

### Luxol Fast Blue (LFB) staining

A series of sections were stained with 1% Luxol Fast Blue (LFB) in 95% ethanol, sealed overnight at 60 °C, washed with distilled water, and placed in 95% alcohol for 10 min. Differentiation was performed with 0.05% lithium carbonate for 10 s and 70% alcohol solution for 20 s. These last two steps were repeated until the gray and white matter were clearly observed under a light microscope. Thereafter, the slides were washed and counterstained with preheated cresyl violet solution at 55 °C for 1 min. The sections were then dehydrated and sealed with neutral gum.

### Image acquisition

Sections were examined and photographed with a Zeiss LSM800 confocal microscope supported by Zen software (2.6 system). Confocal images were then processed using Fiji (ImageJ Version 2.0.0-rc-43/1.51n). All image analyses were performed in a blinded manner to avoid analysis bias. For analyses and bright field image acquisition of LFB staining, 8-bit polychromatic TIFF images of the regions of interest were taken with the same setting for all slides with a Qimaging camera (Qcapture program, version 2.9.10) attached to a Nikon microscope (C-80) with the same gain/exposure settings for every image. Quantification of CD68 + and GFP + particle perimeters was performed using threshold/analyze particle features in Fiji. The background was subtracted from the images, and intensities were equally adjusted within each set of experiments.

### Data analysis

The experimenters were blinded to all groups for the quantifications. The results are expressed as the mean ± SEM (standard error of the mean). Data distribution was analyzed by the Shapiro–Wilk normality test, and Brown–Forsythe was used to check the homogeneity of variance for ANOVA. All data presented in the manuscript passed both tests and were analyzed as normally distributed and with equal variances. Descriptive and inferential statistics were applied to the data using GraphPad Prism version 8.01 (San Diego, CA, United States). P values less than 0.05 (*p* < 0.05) were considered to be statistically significant. Significance is indicated by ^∗^*p* < 0.05; ^∗∗^*p* < 0.01; ^∗∗∗^*p* < 0.001, and ^∗∗∗∗^*p* < 0.0001. All panels were assembled using Adobe Photoshop CC 2018 (version 19.1.0) and Adobe Illustrator CC 2018 (version 23.0.1).

## Results

### Brain levels of melatonin and cortisol are affected by light manipulation

To manipulate endogenous circadian melatonin and cortisol, mice were kept in constant light (CL) or constant darkness (CD) for 3 days followed by a day of recovery using a normal day/night cycle, which was repeated for 5 cycles for a total of 20 days (Fig. 1a). Exogenous melatonin or luzindole was used as an agonist or antagonist of melatonin receptors, respectively **(**Fig. 1a), for the same study period. The results showed that feeding mice CPZ for 5 weeks caused a 23% increase (*p* > 0.05) in brain levels of melatonin in vehicle mice compared to healthy control mice (20.31 ± 0.87 vs. 16.52 ± 0.60 pg/mg/brain; Fig. 1b), whereas cortisol levels did not change compared to controls (0.38 ± 0.02 vs. 0.36 ± 0.01 pg/mg/brain; *p* > 0.05; Fig. 1c). On the other hand, manipulating the circadian rhythm in the constant light (CL) group caused a 33% reduction (*p < 0.05) in melatonin (13.41 ± 0.88 vs. 20.31 ± 0.87 pg/mg/brain;) and a significant (****p < 0.0001) 82% increase in cortisol (0.69 ± 0.08 vs. 0.38 ± 0.02 pg/mg/brain) levels compared to vehicle. However, constant darkness (CD) caused the opposite effect, with a significant (****p < 0.0001) increase in melatonin (33.44 ± 0.32 vs. 20.31 ± 0.87 pg/mg/brain) and an inhibitory effect (*p < 0.05) on cortisol secretion (0.18 ± 0.01 vs. 0.38 ± 0.02 pg/mg/brain) in comparison to vehicle-treated mice. In contrast, no significant changes in the levels of melatonin and cortisol were observed when melatonin receptors were stimulated with exogenous melatonin or inhibited with the antagonist luzindole (Fig. 1b, c).

### Melatonin receptors are expressed by resident macrophages, oligodendrocytes, and NPCs

**Fig. 2:**
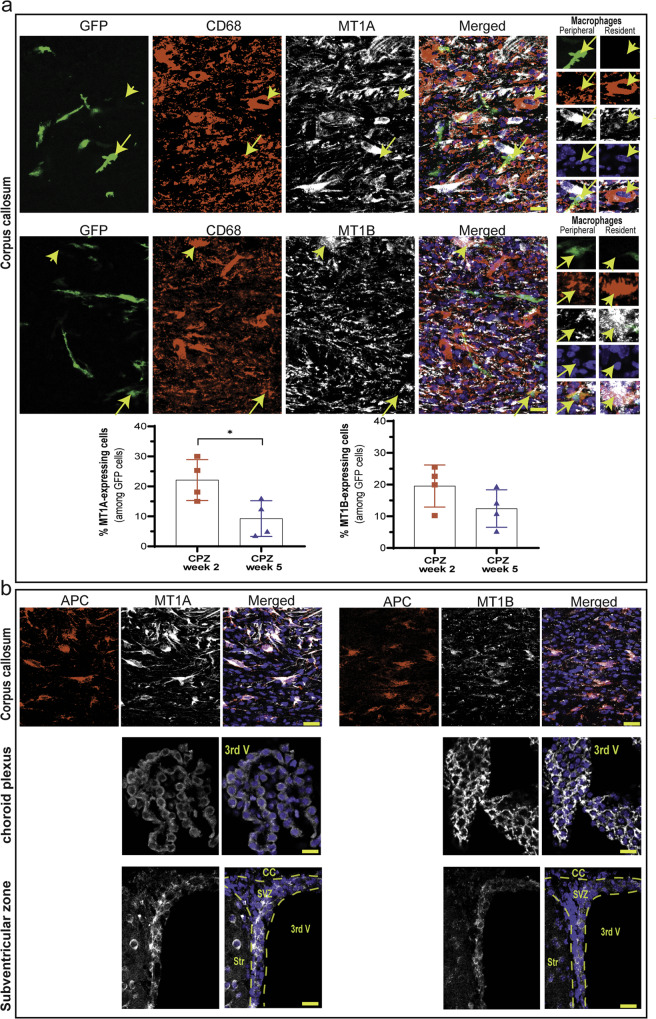
Fluorescent staining to detect the melatonin receptors MT1A and MT1B. **a** Chimeric mice, whose BM-derived monocytes were GFP-tagged, were used to detect the colocalization of GFP (BM-derived cells) and CD68 (monocytes/macrophages). Lack of GFP expression in CD68-positive cells indicates that these cells were brain-resident macrophages (long yellow arrow). However, the expression of GFP indicated infiltrated BM-derived macrophages (short yellow arrow) in the corpus callosum. MT1A and MT1B were individually added to the GFP/CD68 panel for triple staining. MT1A- and MT1B-expressing GFP cells (bone-derived monocytes/macrophages) were quantified 2 and 5 weeks after cuprizone diet feeding. (*n* = 4 mice/group). Significance is indicated by ^∗^*p* < 0.05 using an unpaired *t*-test. **b** Expression of MT1A and MT1B in oligodendrocytes (APC cells) in the corpus callosum (upper panel), choroid plexus (middle panel) and subventricular zone (lower panel). Scale bars, 20 μm.

To study melatonin receptor 1 A (MT1A) and 1B (MT1B) expression in brain resident and BM-derived (peripheral) macrophages in the CNS, these two cell types were discriminated by the use of chimeric mice. Since BM hematopoietic cells are replaced by GFP-expressing cells, any observed GFP cell in the adult CNS is an infiltrated cell. Activated microglia were labeled with CD68, a marker that can be expressed by both monocytes and embryo-derived brain resident macrophages^25^. Therefore, CD68-positive macrophages are considered resident macrophages if they do not colocalize with GFP; however, these cells are considered BM-derived peripheral macrophages if they colocalize with GFP. The results showed that resident macrophages (CD68^+^/GFP^-^) expressed both MT receptors (Fig. 2a; short yellow arrows), whereas peripheral macrophages (CD68^+^/GFP^+^) lacked these receptors (Fig. 2a; long yellow arrows). The detection of GFP in normal mice was not possible because of the absence of infiltration; therefore, cuprizone mice were used, which had high levels of macrophages (CD68) in the corpus callosum. CD68 is localized in cellular, lysosomal, and endosomal membranes. Given that both melatonin receptors are expressed by these brain-resident macrophages, we detected a large dispersion of MT1A and MT1B, which corresponded to the dispersion of CD68.

CD68 cells can be either monocytes or macrophages, while GFP-expressing circulating monocytes can differentiate into macrophages and dendritic cells after infiltrating into the brain. Therefore, some CD68/GFP cells might be undifferentiated peripheral monocytes or at the beginning of their differentiation to macrophages, and these cells may still express MT1A and MT1B. To examine this, we quantified the percentage of MT-expressing CD68^+^ cells among GFP cells in the CC during the course of demyelination. The results showed that 22% of CD68/GFP cells expressed MT1A, while 19% expressed MT1B two weeks after being fed with cuprizone (Fig. 2a; low chart). However, the expression of MT1A and MT1B decreased to 9 and 12%, respectively, after 5 weeks of cuprizone feeding. This result suggests that circulating monocytes in the CNS may express MT receptors as long as they have not fully differentiated into macrophages.

We further studied the expression of melatonin receptors in 3 regions: the corpus callosum, where OLs are damaged in the CPZ model; the choroid plexus, where immune cells can traffic into the CNS; and the SVZ region, where a pool of NSPCs are located. Immunofluorescence staining showed that both MT1A and MT1B were found on APC-expressing OLs in the corpus callosum (Fig. 2b; upper panel), as well as in the choroid plexus (Fig. 2b; middle panel) and SVZ (Fig. 2b; lower panel) regions. In addition to monocytes/macrophages, we examined the expression of MT receptors during the course of demyelination before cuprizone feeding and 2 and 5 weeks after cuprizone administration. The results showed that the expression of MT1A and MT1B in APC oligodendrocytes was not affected by demyelination and that all APC cells expressed these receptors (Supplementary Fig. 1).

### Constant light and inhibiting endogenous melatonin promote oligodendrogenesis in the corpus callosum, mainly due to the maturation of OPCs rather than proliferation

Olig2, a helix-loop-helix transcription factor, is a marker expressed by both immature oligodendrocyte precursor cells (OPCs) and mature OLs. These cells were discriminated by double staining with antibodies against Olig2 and adenomatous polyposis coli (APC), a marker of mature OLs. In addition, since we started BrdU administration at the initiation of the light and treatment conditions (week 3 of a CPZ diet), we only counted newly generated OPCs and OLs that expressed BrdU (OPC: BrdU^+^/Olig2^+^/APC^-^ versus OL: BrdU^+^/Olig2^+^/APC^+^ cells). NSPCs from the SVZ can migrate to the medial and lateral areas of the corpus callosum; however, our quantifications were performed in the middle corpus callosum along the rostrocaudal axis since it is highly vulnerable to CPZ-induced demyelination, whereas the lateral area is more resistant^26^.

**Fig. 3:**
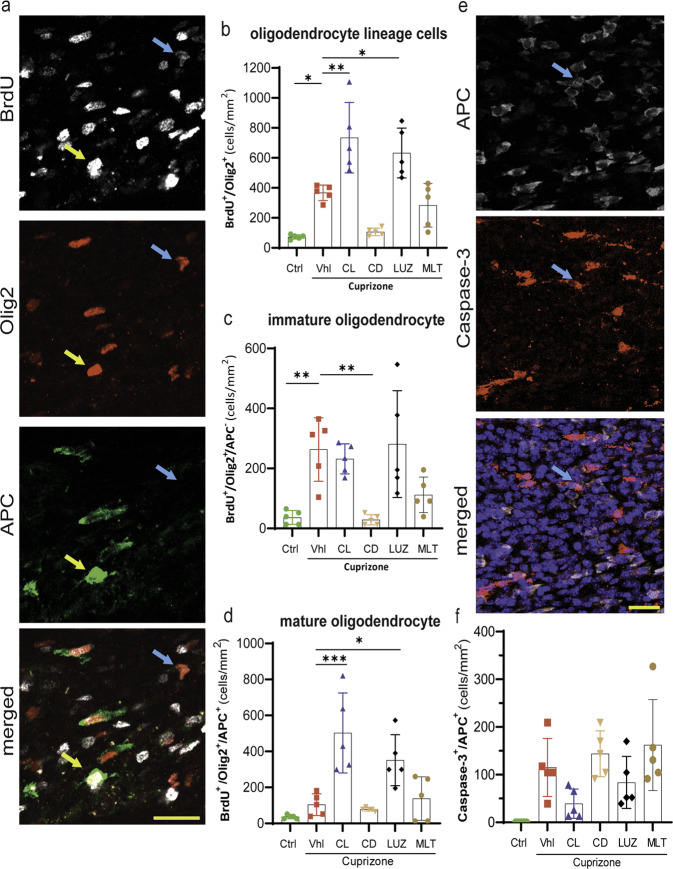
Quantification of the oligodendroglial response to demyelination and treatment in the corpus callosum. **a** Fluorescent staining of newly generated BrdU+ cells. Olig2 is a marker of the oligodendroglial lineage. APC is a marker of mature oligodendrocytes. DAPI staining of the nucleus. Left panel, the blue arrow shows immature OLs, and the yellow arrow shows mature OLs. **b** Quantification of the oligodendroglia lineage (BrdU + /Olig2+ cells), which includes both immature and mature OLs. **c** Quantification of immature OLs (OPCs; BrdU + /Olig2 + /APC- cells). **d** Quantification of mature OLs (BrdU + /Olig2 + /APC + cells). **e** Costaining of the apoptosis marker Caspase-3 and the oligodendrocyte marker APC. Right panel: The arrow indicates Caspase-3 colocalization with APC. **f** Quantification of the mean number of Caspase-3 + /APC + cells. All quantifications were performed in the middle corpus callosum along the rostrocaudal axis. (*n* = 5 mice/group; 3 slides per mouse). Significance is indicated by ^∗^*p* < 0.05; ^∗∗^*p* < 0.01; and ^∗∗∗^*p* < 0.001. Scale bars, 20 μm.

The results showed a significant (*p < 0.05) increase in newly generated oligodendroglia cells (BrdU^+^/Olig2^+^ cells), including both OPCs and OLs, in vehicle mice (369 ± 23) compared to control mice (78 ± 8). Discrimination between the two cell types among newly generated Olig2 cells demonstrated that this increase occurred mainly in OPCs (Fig. 3a). The OPC population (BrdU^+^/Olig2^+^/APC^-^ cells) was significantly (**p < 0.05) higher in vehicle mice (264 ± 48) than in control mice (37 ± 10), while the OL population (BrdU + /Olig2 + /APC + cells) was higher in vehicle mice (105 ± 27) than in control mice (37 ± 5), without reaching statistical significance (Fig. 3b–d).

In addition, Olig2+ cells showed a further significant increase due to constant light (737 ± 106) or luzindole (636 ± 74) in comparison to vehicle mice (369 ± 23) (**p < 0.01 vs. *p < 0.05, respectively) (Fig. 3b); however, this increase was mainly in mature OLs but not immature OPCs. Immature OPCs did not increase in response to constant light (233 ± 22) or luzindole (282 ± 80) in comparison to those in the vehicle group (264 ± 48) (Fig. 3c); however, the maturation of OPCs into OLs increased significantly in response to constant light (505 ± 100) or luzindole (353 ± 64) (Fig. 3d) in comparison to that in the vehicle group (105 ± 27) (***p < 0.001 vs. *p < 0.05, respectively) (Fig. 3b–d).

In contrast, constant darkness (107 ± 12) and melatonin (285 ± 65) caused an insignificant reduction in total Olig2+ oligodendroglia compared to that in the vehicle group (367 ± 105), and the difference induced by constant darkness was very close to significance (*p* = 0.051). However, this reduction was mainly in immature OPCs but not mature OLs. Immature OPCs were significantly (**p < 0.01) reduced by constant darkness (29 ± 8) but not by melatonin (112 ± 27) in comparison to that in the vehicle group (264 ± 48). In contrast, mature OLs did not significantly change in response to constant darkness (78 ± 4) or melatonin (139 ± 54). It seems that constant darkness and exogenous melatonin reduces OPC proliferation (Fig. 3b–d).

### Increased oligodendrogenesis is not due to a reduction in apoptosis

To investigate whether the increase in OLs induced by constant light or luzindole was due to increased maturation of OPCs or reduced apoptosis, the number of apoptotic OLs was quantified using Caspase-3, which colocalized with the OL marker APC (Fig. 3e). The results showed that apoptotic OLs (Caspase-3 + /APC + cells) were decreased in the constant light group (39 ± 14) in comparison to the vehicle group (115 ± 27). However, this decrease in apoptosis did not reach statistical significance, possibly because of ongoing apoptosis due to the CPZ diet. On the other hand, none of the luzindole (84 ± 24), constant darkness (144 ± 22), or melatonin (162 ± 43) groups showed a significant change in apoptosis compared to the vehicle group (115 ± 27) (Fig. 3f).

### Constant darkness and exogenous melatonin therapy suppress NSPC proliferation

**Fig. 4:**
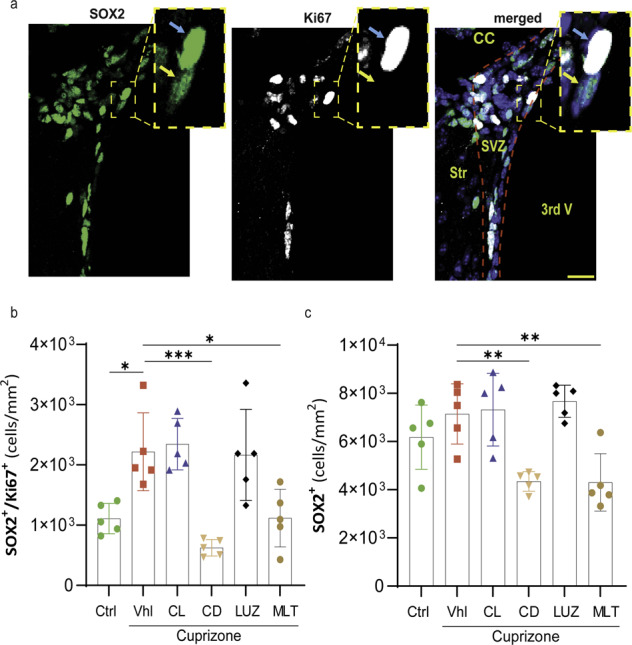
Quantification of NSPC responses to demyelination and treatment in the SVZ. **a** Fluorescent staining of the NSPC marker SOX2, the proliferation marker Ki67, and DAPI. The blue arrow indicates proliferative SOX2, and yellow shows quiescent NSPCs. **b** Quantification of the number of proliferative NSPCs (SOX2 + /Ki67 + ). **c** Quantification of the number of NSPCs (SOX2 + ), which included both quiescent and proliferative NSPCs. All quantifications were performed in the SVZ region on 25 μm-thick sections (*n* = 5 mice/group; 3 slides per mouse). Significance is indicated by ^∗^*p* < 0.05, ^∗∗^*p* < 0.01, and ^∗∗∗^*p* < 0.001. Scale bars, 20 μm.

Given that NSPCs in the SVZ region are among the oligodendroglia pools in adults and that melatonin receptors are expressed by SVZ cells, we investigated the effect of light and melatonin on NSPC responses to CPZ-induced demyelination. The results showed that the proliferation of NSPCs (SOX2^+^/Ki67^+^ cells) in the SVZ region was significantly (*p < 0.05) increased in the vehicle CPZ group (2219 ± 289) in comparison to the control group (1109 ± 112) **(**Fig. 4a, b). This increase in NSPC proliferation was not altered by constant light (2344 ± 191) or luzindole (2164 ± 339); however, constant darkness (625 ± 62, ***p < 0.001) and melatonin (1117 ± 214, *p < 0.05) significantly inhibited this proliferation (Fig. 4b).

Given that uncontrolled proliferation can deplete the NSPC pool, SOX2-expressing NSPCs were counted in the SVZ region (Fig. 4c). The results showed that SOX2-expressing cells were increased by CPZ in the vehicle group (7148 ± 559) compared to the control group (6180 ± 596), but the difference was not significant. Moreover, constant light (7320 ± 673) or luzindole (7672 ± 298) did not affect the number of SOX2-expressing NSPCs. In contrast, constant darkness (4344 ± 182; **p < 0.01) or melatonin (4305 ± 534; **p < 0.01) significantly reduced the number of SOX2-expressing NSPC cells compared to that in vehicle mice (7148 ± 559) (Fig. 4c). Therefore, increased proliferation of SOX2-expressing cells did not deplete the NSPC pool in groups of constant light and luzindole.

### Constant light and luzindole promote the recruitment of OPCs to the site of demyelination and the restoration of myelin sheaths

**Fig. 5:**
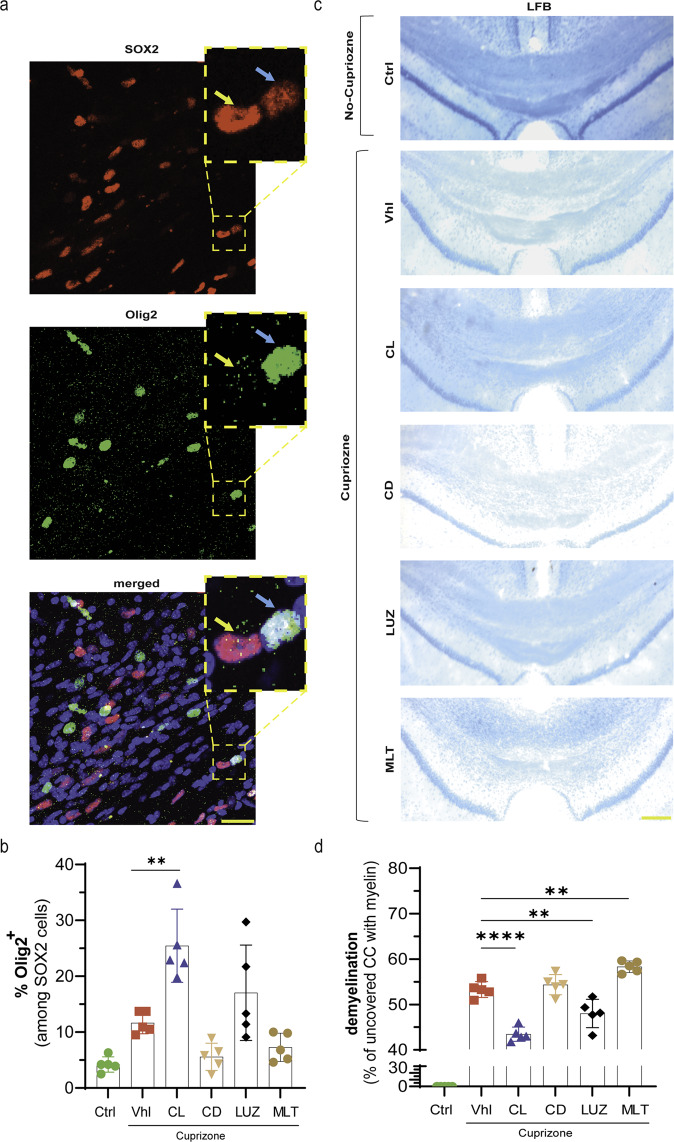
Olig2 recruitment to the demyelinated site and restoration of the myelin sheath. **a** Fluorescent staining of Olig2, an oligodendroglia marker, and SOX2, which is expressed by Olig2 cells, increased the recruitment of Olig2+ cells to the site of injury. **b** Quantification of the frequency of SOX2-expressing Olig2 cells (SOX2 + /Olig2 + ). The blue arrow indicates SOX2 + /Olig2 + , and yellow indicates SOX2 + /Olig2^-^. **c** LFB staining of the corpus callosum to measure the extent of demyelination. **d** Quantification of the percentage of the uncovered area of the corpus callosum, which indicates the demyelinated area. Olig2, SOX2 and LFB quantifications were performed in the same area of the corpus callosum. The blue arrow shows Olig2-expressing SOX2 cells, and the yellow arrow shows SOX2 lacking Olig2 expression (*n* = 5 mice/group; 3 slides per mouse). Significance is indicated by ^∗∗^*p* < 0.01 and ^∗∗∗^*p* < 0.0001. Scale bars, 20 μm in **a**; 100 μm in **c**.

It has already been reported that SOX2 is expressed at different ratios by OPCs and astrocytes in the CPZ model, probably due to the time or site of assessment following the induction of demyelination^27^. Researchers also showed that an increase in SOX2 was crucial for the differentiation and recruitment of new OPCs to the site of injury. Here, we investigated whether increasing SOX2 expression in the SVZ was associated with an increase in the number of OPCs in the corpus callosum, which would lead to the differentiation and recruitment of OPCs to the site of demyelination (Fig. 5a). Double immunostaining with SOX2 (nucleus) and Olig2 showed that the percentage of SOX2-expressing Olig2 cells increased more than 2-fold in vehicle mice (11.7 ± 0.9) compared to control mice (4.2 ± 0.6), but the difference was not significant (*p* = 0.16). This change was further increased in the constant light (25.5 ± 2.9, **p < 0.01) or luzindole (17.0 ± 3.8, *p* > 0.05) groups. However, neither constant darkness (5.6 ± 1.1, *p* > 0.05) nor melatonin (7.3 ± 1.1, *p* > 0.05) caused a significant decrease in the percentage of Olig2 + /SOX2 + cells in the corpus callosum (Fig. 5b).

This increase in the recruitment of Olig2 cells to the demyelination site was further examined by measuring the restoration of myelin sheaths by LFB staining (Fig. 5c). Myelin regeneration and a reduction in the demyelinated area were expected if Olig2 cells were recruited to the demyelination site and the maturation of OLs was well established. The results showed that the demyelinated area was significantly reduced by constant light (43.4 ± 0.7%; ****p < 0.0001) and luzindole (48.0 ± 1.4%; **p < 0.01) in comparison to that in vehicle mice (53.3 ± 0.8%). In contrast, melatonin caused a significant increase (56.7 ± 0.6%; **p < 0.01) in the demyelination area but not in the constant darkness group (54.4 ± 1.0%; *p* > 0.05) (Fig. 5d).

### Constant light increases patrolling monocytes, phagocytosis, and the clearance of myelin debris

**Fig. 6:**
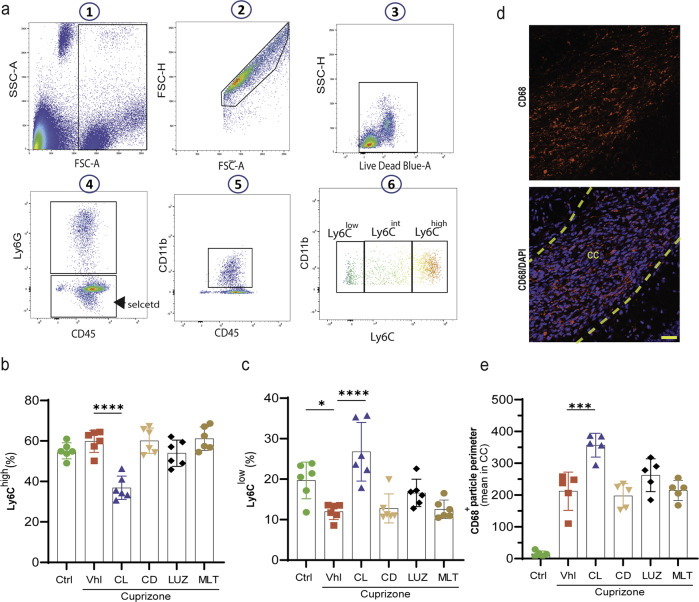
Analysis of monocyte subtypes by FACS and phagocytosis. **a** Gating strategy for monocytes. After gating for live single cells, neutrophils were excluded from populations (CD45 + /Ly6G-) and then gated for CD11b + /CD45 + populations. These cells were further gated with CD11b + /Ly6C to analyze the percentage of Ly6Clow, Ly6Cint, and Ly6Chigh cells. **b** Percentage of classical inflammatory monocytes (Ly6Chigh). **c** Percentage of patrolling monocytes (Ly6Chigh). **d** Fluorescent staining of the phagocytosis marker CD68. **e** The corresponding quantifications. All quantifications were performed on peripheral blood samples collected at the same time (*n* = 5 mice/group). Significance is indicated by **p < 0.01 and ****p < 0.0001. Scale bars, 20 μm.

Considering that classical monocytes (Ly6C^high^) play a proinflammatory role, whereas nonclassical monocytes (Ly6C^low^) can patrol the vasculature and perform phagocytosis to prepare for remyelination^28^, these monocytes were examined in the circulating blood using FACS (Fig. 6a). The results showed that feeding mice CPZ did not affect the population of classical Ly6C^high^ monocytes (59.8 ± 2.2) in comparison to the control group (55.1 ± 1.6) (Fig. 6b). However, constant light (36.9 ± 2.4) caused a significant (****p < 0.0001) decrease in these monocytes compared to that in the vehicle group (59.8 ± 2.2). On the other hand, constant darkness (60.1 ± 2.6), luzindole, (53.9 ± 2.6), or melatonin (61.2 ± 2.4%) failed to affect the percentage of the Ly6C^high^ cell population (Fig. 6b).

Given that classical Ly6C^high^ monocytes can switch to patrolling Ly6C^low^ monocytes^28^, we further quantified this population (Fig. 6c). The data showed that Ly6C^low^ monocytes were significantly (*p < 0.05) decreased in vehicle mice (12.0 ± 0.8) in comparison to control mice (19.7 ± 1.8). Importantly, this reduction was restored by constant light (26.8 ± 2.9), which caused a significant (****p < 0.0001) increase in the level of Ly6C^low^ in comparison to vehicle, highlighting that light could switch the classical monocytes into patrolling monocytes. However, neither luzindole (16.6 ± 1.4), constant darkness (12.8 ± 1.5), nor melatonin (12.6 ± 0.9) affected the percentage of Ly6C^low^ monocytes compared to vehicle (Fig. 6c).

Since an increase in patrolling monocytes is accompanied by an increase in phagocytosis, we examined the expression of CD68, a marker of phagocytosis, in the corpus callosum (Fig. 6d). The results showed that phagocytosis was significantly (****p < 0.0001) increased in vehicle mice (212 ± 27) in comparison to control mice (15 ± 4), which was significantly (***p < 0.001) accentuated by constant light (357 ± 17) therapy. However, none of the mice treated with luzindole (262 ± 23), constant darkness (198 ± 17) or melatonin (215 ± 14) showed any significant changes compared to vehicle mice (Fig. 6e).

### Immune cell infiltration into the CNS is exacerbated by constant darkness

**Fig. 7:**
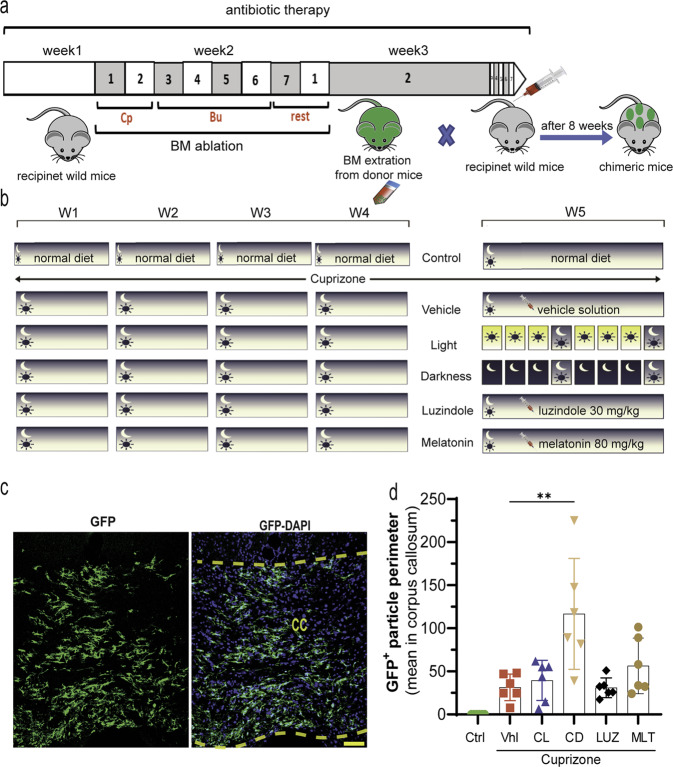
The infiltration rate in the CNS. **a** Schematic illustration of establishing chimeric mice and (**b**) the protocol for the treatments in chimeric mice (antibiotic, Septra; Busulfan, Bu; Cyclophosphamide, Cp). **c** A representative example of GFP-tagged cells infiltrating the brain in the corpus callosum. **d** The corresponding quantifications. (*n* = 5 mice/group; 3 slides per mouse). Significance is indicated by ^∗∗^*p* < 0.01, ^∗∗∗^*p* < 0.001, and ^∗∗∗∗^*p* < 0.0001. Scale bars, 20 μm.

In addition to the blood-brain barrier (BBB), which is formed by brain endothelial cells (ECs), the blood-cerebrospinal fluid barrier (BCSFB), which is formed by epithelial cells of the choroid plexus (CP), plays a crucial role in infiltration into the CNS^29^. Despite the very limited studies on immune cell infiltration into the CNS in the CPZ demyelination model, Shelestak et al. showed that the BBB loses its integrity 3 days after starting the CPZ diet, when there is no demyelination yet.^30^ In addition, melatonin has been reported to protect BBB integrity^31^. However, our results showed that melatonin receptors are expressed in the choroid plexus where the BCSFB is established. Thus, we hypothesized that altering the endogenous/exogenous levels and function of melatonin receptors may affect the infiltration rate. We used BM-chimeric GFP mice (protocol summarized in Fig. 7a, b) and found that 5 weeks of a CPZ diet resulted in a massive infiltration of systemic immune cells (31.3 ± 6.3) compared to that in control mice, in which no GFP was detected. It is worth noting that the treatment was started at the beginning of Week 5, when disruption of the immune cell barrier in the brain and demyelination are at their peaks, which allows us to better analyze the potency of our treatments in ameliorating or exacerbating infiltration. Our results showed that constant darkness (116.8 ± 26.3) significantly (**p < 0.01) exacerbated immune cell infiltration; however, melatonin (56.4 ± 13.2; *p* > 0.05), constant light (39.4 ± 9.6; *p* > 0.05) or luzindole (30.9 ± 4.7; *p* > 0.05) failed to reduce GFP levels in comparison to those in the vehicle group (Fig. 7c, d).

## Discussion

The present study revealed that induction of demyelination by cuprizone is followed by a slight increase in melatonin levels in the brain when melatonin is assessed at the end of the protocol. To rule out any bias, we believe that melatonin measurements are needed during the various time points of the experimental procedure to uncover any increase in melatonin during the course of the disease. In addition, constant light maintained melatonin in the brain at a low level, while constant darkness substantially increased it. In contrast, the administration of exogenous melatonin or luzindole had no effect on melatonin levels. These findings demonstrated the capability of our protocol to chronically reduce or increase melatonin by the pineal gland, which is the only light-dependent source of melatonin synthesis.

Although there has been no report regarding the effect of cuprizone as a copper chelating agent on melatonin synthesis, Parmar and Daya showed that treating pineal organ cultures with copper inhibited the key enzyme in melatonin synthesis^32^. We believe that chelating copper with cuprizone eliminates the regulatory effects of this enzyme, leading to an increase in melatonin synthesis.

On the other hand, constant light was associated with a sharp increase in brain cortisol levels, while constant darkness had the opposite effect. Previous studies reported that continuous light at night considerably increased the level of corticosterone in mice^33^. Glucocorticoids, such as cortisol, can inhibit the recruitment of immune cells to the site of injury or act as immunosuppressants^34^. In MS, it has been shown that rats that were chronically exposed to stress for 3 weeks had reductions in EAE incidence and myelin loss and lower leukocyte infiltration into the CNS^35^. In fact, corticosteroid therapy, which is designed to mimic the anti-inflammatory effect of naturally released cortisol, is widely used for MS therapy. Therefore, we hypothesized that the simultaneous increase in cortisol (a powerful immunosuppressive hormone) and the chronic inhibition of peak melatonin secretion at night can maintain melatonin at sufficient levels to exert its beneficial effects as an immunoregulator rather than an immunoenhancer in MS. This could be considered an invasive strategy for suppressing the deleterious effects of immune cell attack on myelin or increasing the oligodendrogenesis process required for recovering lost myelin. Melatonin could reduce cortisol levels^36^, which may further explain why a reduction in melatonin in the constant light group was associated with a sharp increase in cortisol levels.

On the other hand, most studies on MS administered melatonin in the evening before the night began, which causes disequilibrium in the circadian rhythm^37^. A nocturnal increase in melatonin causes a more than 40% decrease in circadian core body temperature^38^. However, evening administration of melatonin leads to a longer and earlier reduction in core body temperature, which suppresses the immune system and plays an anti-inflammatory role^39^. In fact, evening melatonin therapy plays a beneficial role in MS indirectly, but studies are needed to determine its effect when melatonin levels are boosted. In this study, treatment was performed during the day, when melatonin is at its lowest level, to boost diurnal melatonin, in addition to its effect at night. Based on our previous study^40^, placing mice in constant light for 10 days failed to diminish serum melatonin levels, possibly due to melatonin synthesis by compensatory mechanisms of pineal-independent sources that occur after the first days of darkness. Hongas et al.^41^ used constant light or darkness therapy on the day of the induction of spinal cord injury (SCI) and showed that melatonin levels in the cerebrospinal fluid (CSF) increased on Day 3 post SCI in the group exposed to constant darkness; however, this elevation started to abate after 3 days and approached control levels on Day 7 of darkness. This finding further emphasized that long-term light manipulation could not maintain melatonin at low or high levels. Our new protocol of light manipulation aimed to minimize the negative feedback of the pineal gland or other organs in response to the continuous increase in melatonin in the dark group and the compensatory mechanisms of other sources of melatonin synthesis in response to the continuously decreased melatonin levels in constant light.

Since melatonin affects cell physiology through its receptors, we investigated the expression of melatonin receptors 1 A and 1B (MT1A and MT1B) in the corpus callosum. We detected both receptors in OLs in the corpus callosum. Although feeding mice cuprizone causes demyelination, the number of immature OLs but not mature OLs increases as a self-regeneration mechanism in mice. Our data showed that constant darkness markedly suppressed OPC proliferation compared to that in untreated CPZ mice. In contrast, neither constant light nor luzindole therapy boosted OPC proliferation. It seems that the CPZ-induced pathological condition exerted its maximum stimulatory effects on OPC proliferation; thus, constant light and luzindole failed to stimulate further proliferation, which is expected to lead to NSPC pool depletion. Interestingly, although the increased number of immature OPCs in untreated CPZ mice failed to become mature OLs, constant light and luzindole significantly protected the maturation of OPCs to OLs. This was further confirmed by LFB staining, which showed that constant light and luzindole not only increased the maturation of OPCs but also completed the remyelination process and myelin sheath reconstruction.

Since we revealed that MT1A and MT1B were expressed in the SVZ region and considering that previous reports showed that OPCs in the corpus callosum can originate from the SVZ under demyelinating conditions^42,43^, we observed that CPZ induces a sharp increase in the proliferation of SVZ NSPCs, most likely because of the response of the SVZ to demyelination and inflammation to recover the damaged OLs. However, neither constant light therapy nor luzindole could increase SOX2 activation, which seems to be at its highest level. Since SOX2-expressing cells can migrate and differentiate into neurons, astrocytes, and oligodendrocytes in demyelination sites, we quantified Olig2/SOX2 expression in the corpus callosum, as SOX2 is expressed by new OPCs to contribute to their recruitment to the demyelination site^27,44^. Although the groups treated with vehicle, constant light and luzindole had similar levels of SOX2 activation in the SVZ, only constant light increased Olig2-expressing SOX2 cells in the corpus callosum, suggesting that a significant portion of SOX2-expressing cells had differentiated into OPCs.

**Fig. 8:**
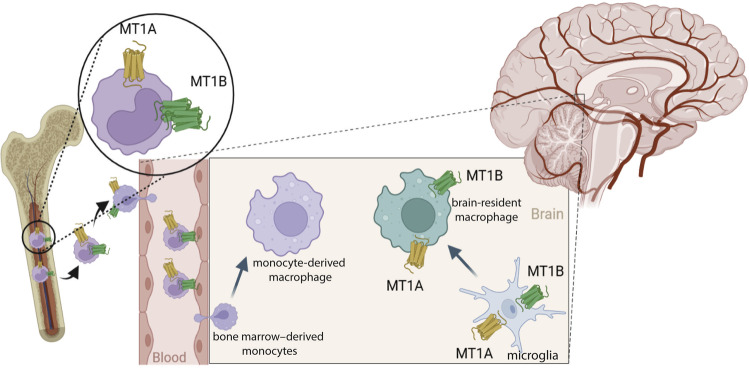
Schematic illustration of melatonin receptor (MT1A and MT1B) expression by monocytes/macrophages. Monocytes express melatonin receptors in the BM and the circulation. However, monocytes lose this expression after infiltrating the CNS and differentiating into macrophages. On the other hand, microglia, and brain-resident macrophages express melatonin receptors, while BM-derived macrophages lack this expression in demyelinated mice.

From an immunological perspective, we used chimeric mice and observed that BM-derived microglia/macrophages (CD68^+^/GFP^+^ cells) in the CNS did not express MT1A or MT1B; however, brain-resident microglia/macrophages (CD68^+^/GFP^-^ cells) expressed both receptors. In support of this finding, an early in vitro study in 1998 using binding experiments of 2-[^125^I]iodomelatonin showed that fresh and 1-day cultured human monocytes expressed high levels of melatonin receptors, which was lost after 3 days and 15 days of culture because of the maturation or differentiation of monocytes, respectively. The authors also showed that the U937 monocytic cell line did not express melatonin receptors^45^. In contrast, another group used the same binding technique on purified U937 cell nuclei and membranes and reported that these cells expressed MT1 only^46^. However, the findings regarding melatonin receptor expression in macrophages but not monocytes are still controversial. It seems that while monocytes in the circulation express melatonin receptors, they lose this expression upon differentiation into macrophages in the CNS of demyelinated mice. The current knowledge and our findings regarding melatonin receptor expression in macrophages are illustrated in Fig. 8.

The involvement of monocytes in the CPZ model has rarely been studied; therefore, we further investigated the response of monocytes to our various manipulations. Our data revealed that while the CPZ diet significantly decreased classical anti-inflammatory monocytes (Ly6C^low^), constant light and luzindole restored this expression, unlike constant darkness or melatonin. This finding further highlights the existence of melatonin receptors in monocytes; however, more experiments are needed to determine the exact expression of the various melatonin receptors in different types of monocytes.

Our data revealed that both melatonin receptors MT1A and MT1B are expressed in the choroid plexus, which is one of the barriers to immune cell infiltration into the CNS. Chimeric mice treated for 1 week, and at Week 5 of the CPZ diet, we demonstrated that monocyte infiltration into the CNS was exacerbated by constant darkness and melatonin but not constant light and luzindole. Since even one week of treatment plays a deleterious role in infiltration, this experiment should be performed at the time of CPZ diet initiation, or even before, as well as under chronic treatment conditions. Furthermore, characterization of the function of peripheral or resident macrophages in the corpus callosum revealed that constant light and luzindole increased the phagocytotic activity of macrophages. Since BM-derived macrophages in the CNS may not express melatonin receptors, this finding suggests that these effects on the phagocytotic activity of macrophages are mediated by brain resident microglia/macrophages. Furthermore, a study on the murine macrophage cell line RAW264.7 showed that melatonin inhibits phagocytosis, as visualized and quantified by CFSE staining of E. coli^47^.

While the beneficial effect of melatonin has been reported in a wide range of diseases, it should be noted that this molecule has pleiotropic effects. In fact, its immunoenhancing or immunosuppressive roles in autoimmune disease are still controversial, and some studies have reported a deleterious role in MS models. In addition, case studies reported that using picoTesla magnetic fields improved disability in MS patients, which was accompanied by a reduction in melatonin levels, possibly by downregulation of its immunoenhancing effects^48^. This deleterious role of melatonin in MS was also proposed by Kuklina et al.^49^ and Constantinescu et al.^2^ Moreover, in cultured human monocytes, melatonin showed a high immunoenhancing effect by activating these monocytes and increasing IL-1α and IL-1β^45^. Furthermore, another study showed the potential of melatonin to increase IFN-γ, IL-2, and IL-6 in circulating human CD4 + cells through the melatonin nuclear receptor^50^. Finally, an investigation on Parkinson’s disease showed that melatonin therapy exacerbated motor function and associated behavioral impairment, while an improvement in these abnormalities was achieved by suppressing endogenous melatonin synthesis by pinealectomy or constant light^51^.

Although current medications ameliorate or control relapsing-remitting MS (RRMS), there is still no FDA-approved medication for primary-progressive MS (PPMS) or secondary-progressive MS (SPMS). Patients from the latter categories do not respond to medications prescribed to treat RRMS. Therefore, instead of using the EAE model, which corresponds to RRMS, we took advantage of the cuprizone model, which mimics several characteristics of progressive MS. This allowed us to demonstrate that suppressing natural melatonin and increasing the immunosuppressant property of cortisol could be a new approach for improving the disease course in progressive MS.

Overall, this study highlights the importance of inspecting melatonin levels in MS patients. Constant light manipulation only affected the increase in melatonin at night in cuprizone mice, which is an animal model with a tendency to have increased melatonin levels. However, other light-independent sources of melatonin synthesis, such as the BM, thymus, lymphocytes, and gastrointestinal tract, provide considerable melatonin levels that seem to be sufficient for mediating normal physiological activity. Current reports regarding melatonin do not consider that MS patients have different dietary habits that could be high or low in melatonin and different lifestyles, such as light at night, night shift work, or looking at LED screens at night, all of which interrupt the circadian rhythm of melatonin. In fact, melatonin administration must be prescribed based on the circadian rhythm of each patient to avoid melatonin overdose due to high endogenous and exogenous levels. Therefore, melatonin seems to have its optimal beneficial effect when it is at its physiological concentration or when its immunoenhancing effects resulting from its peak secretion at night is inhibited. Finally, one limitation to this study was the need to measure melatonin levels in patients before and during exogenous melatonin therapy. It is important that the findings of this study be translated to a clinical study by examining MT receptor expression in circulating monocytes or in microglia/macrophages of postmortem MS patients.

## Supplementary information

Supplementary files

## Data Availability

The data that support the findings of this study are available from the corresponding author upon reasonable request.
