# Author Correction: Inhibiting nighttime melatonin and boosting cortisol increase patrolling monocytes, phagocytosis, and myelination in a murine model of multiple sclerosis

**DOI:** 10.1038/s12276-023-00948-8

**Published:** 2023-02-15

**Authors:** Majid Ghareghani, Vincent Pons, Nataly Laflamme, Kazem Zibara, Serge Rivest

**Affiliations:** 1 Neuroscience Laboratory, CHU de Québec Research Center, Department of Molecular Medicine, Faculty of Medicine, Laval University, 2705 Laurier Boul., Québec City, QC G1V 4G2 Canada; 2 PRASE and Biology Department, Faculty of Sciences-I, Lebanese University, Beirut, Lebanon

**Keywords:** Neuroimmunology, Multiple sclerosis

Correction to: *Experimental & Molecular Medicine* 10.1038/s12276-023-00925-1, published online 13 January 2023

After online publication of this article, the authors noticed few errors in the Fig. 1b and Fig. 1c that were inadvertently introduced owing to a technical error. The unit for melatonin’s concentration is (pg/mg brain) rather than (ng/mg brain). The concentrations of all groups is divided by 100 to report the exact values as 16.52 ± 0.60 (pg/mg brain; control) rather than 1652 ± 60 (ng/mg brain; control); 20.31 ± 0.87 (pg/mg brain; vehicle) rather than 2031 ± 87 (ng/mg brain; vehicle); 13.41 ± 0.88 (pg/mg brain; constant light) rather than 1341 ± 88 (ng/mg brain; constant light); 33.44 ± 0.32 (pg/mg brain; constant darkness) rather than 3344 ± 32 (ng/mg brain; constant darkness); 18.79 ± 1.12 (pg/mg brain; luzindole) rather than 1879 ± 112 (ng/mg brain; luzindole); 24.70 ± 0.99 (pg/mg brain; melatonin) rather than 2470 ± 99 (ng/mg brain; melatonin). Figure 1C, the value of all the groups is multiplied by 0.05, a missed factor in the calculation, to report the result as 0.36 ± 0.01 rather than 7.2 ± 0.3 (pg/mg brain; control); 0.38 ± 0.02 rather than 7.6 ± 0.4 (pg/mg brain; vehicle); 0.69 ± 0.08 rather than 13.8 ± 1.7 (pg/mg brain; constant light); 0.18 ± 0.01 rather than 3.6 ± 0.2 (pg/mg brain; constant darkness); 0.39 ± 0.02 rather than 7.9 ± 0.4(pg/mg brain; luzindole); 0.26 ± 0.02 rather than 5.4 ± 0.5; (pg/mg brain; melatonin). The Fig. 1b and 1c are replaced with correct versions.
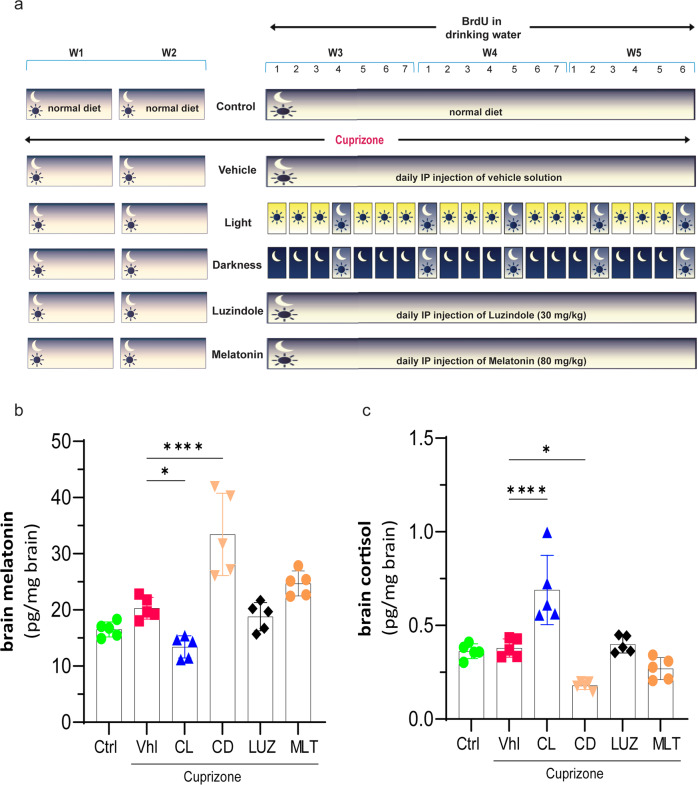


Authors also corrected the following typo in section of results: “Immature OPCs were significantly (**p < 0.01) reduced by constant darkness (29 ± 8) but not by melatonin (112 ± 27) in comparison to that in the vehicle group (264 ± 48 vs 105 ± 27, respectively).” Is replaced by “Immature OPCs were significantly (**p < 0.01) reduced by constant darkness (29 ± 8) but not by melatonin (112 ± 27) in comparison to that in the vehicle group (264 ± 48).”

The authors apologize for any inconvenience caused.

The original article has been corrected.

