# Supplementary material for: Inhibiting nighttime melatonin and boosting cortisol increase patrolling monocytes, phagocytosis, and myelination in a murine model of multiple sclerosis
Source: Exp Mol Med. 2023 Jan 13;55(1):215–27. doi: 10.1038/s12276-023-00925-1 (PMC9898548; doi:10.1038/s12276-023-00925-1)
Supplement: Supplementary file 1 — Supplementary files [file 12276_2023_925_MOESM1_ESM.pdf]

Supplementary Table 1: List of antibodies used in this study for tissue immunofluorescent staining and Fluorescence-Activated Cell Sorting (FACS) analysis.

Num	Product	CAS No.	Vendor
1	Rabbit Melatonin Receptor 1A	Orb221456	Boorbyt
2	Rabbit Melatonin Receptor 1B	NLS932	Novus Biologicals
3	Rabbit Anti-Olig2	AB9610	Millipore
4	Mouse Anti-APC (CC1)	OP80	Calbiochem
5	Rat Anti-BrdU	AB6326	Abcam
6	Rat Anti-SOX2	14-9811-82	Invitrogen
7	Rabbit Anti-Ki67	NB110-57147	Novus Biologicals
7	Rabbit Anti-Caspase-3	9664S	Cell Signaling
8	Rat anti Mouse CD68	MCA1957	BIORAD
9	DAPI	D3571	Invitrogen
10	Mouse CD11b, AF700	56-0112-82	Invitrogen
11	Mouse Ly-6C, V450	560594	BDbioscience
12	Mouse Ly6G, PE	551461	BDbioscience
13	Mouse CD45, V500	561487	BDbioscience
14	CD16/CD32 (Mouse BD Fc Block™)	553142	BDbioscience
15	LIVE/DEAD™ Dead Cell Stain Kit	L23105	Invitrogen

Supplementary Table 2: List of chemical products and drugs used in the study.

Num	Product	CAS No.	Vendor
1	Melatonin	sc-207848B	Santa Cruz
2	Luzindole	sc-202700B	Santa Cruz
3	5-Bromo-2′-deoxyuridine (BrdU)	B5002	Millipore
4	Solvent Blue 38 practical grade (LFB)	S3382	Sigma-Aldrich
5	Bis(cyclohexanone)oxaldihydrazone (Cuprizone)	14690	Sigma-Aldrich
6	Mouse Melatonin (MT) ELISA Kit	MBS263465	MyBioSource
7	Mouse Cortisol ELISA Kit	MBS704879	MyBioSource

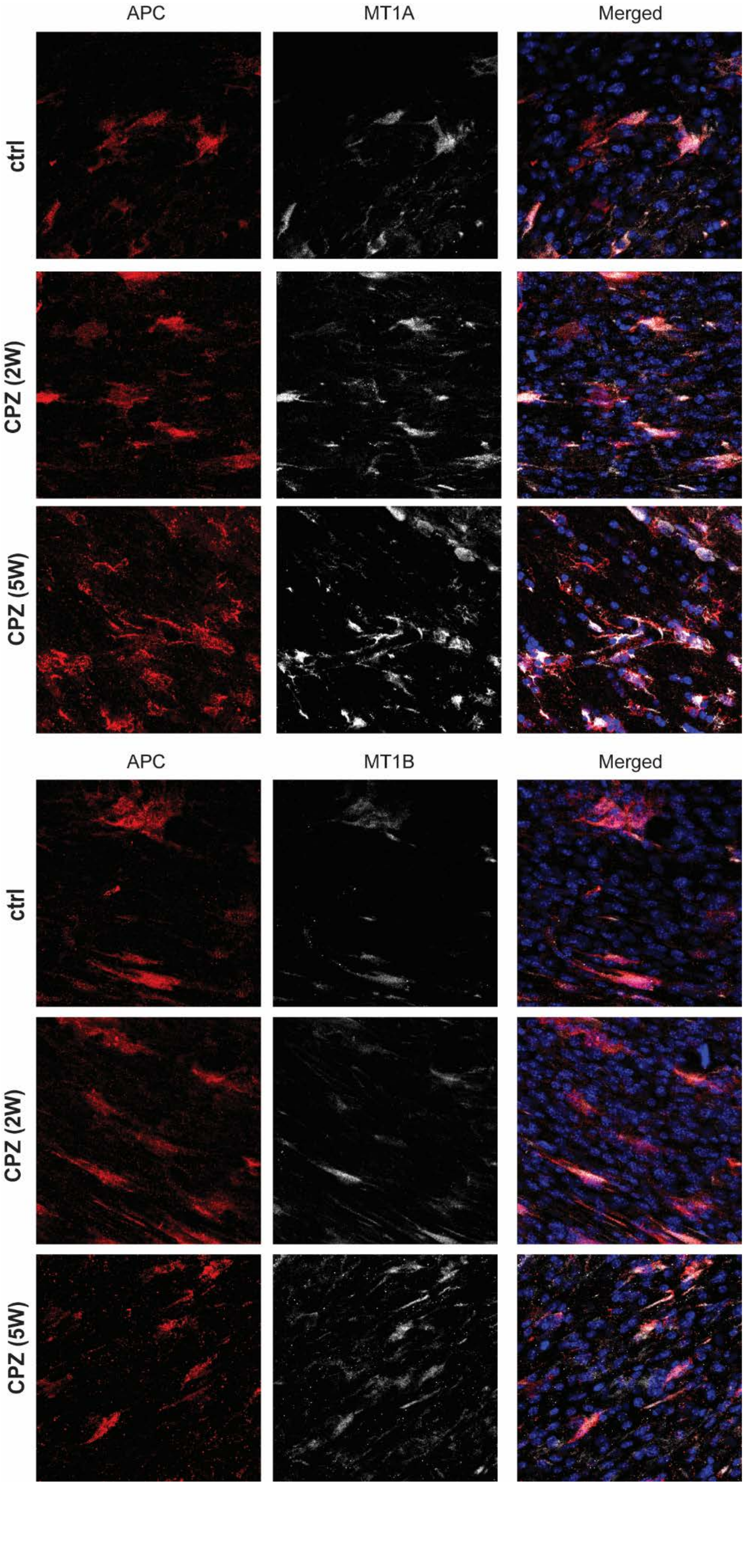
APC
MT1A
Merged
ctrl
CPZ (2W)
CPZ (5W)
APC
MT1B
Merged
ctrl
CPZ (2W)
CPZ (5W)

Supplementary Fig. 1: Representative immunofluorescent images of MT expressing APC-oligodendrocyte. The expression of MT1A and MT1B examined before cuprizone diet, two and five weeks after cuprizone. All APC oligodendrocytes expressed MT receptors at all time points.
